# Unveiling NUSAP1 as a common gene signature linking chronic HBV infection and HBV-related HCC

**DOI:** 10.1007/s12672-024-00922-4

**Published:** 2024-03-05

**Authors:** Jiao Meng, Zhenkun Yang, Xinyi Jiang, Jian Zou

**Affiliations:** Department of Laboratory Medicine, The Affiliated Wuxi People’s Hospital of Nanjing Medical University, Wuxi People’s Hospital, Wuxi Medical Center, Nanjing Medical University, 299 Qingyang Road, Wuxi, 214023 Jiangsu China

**Keywords:** Hepatocellular carcinoma, Chronic HBV infection, NUSAP1, Cell cycle

## Abstract

**Background:**

Hepatitis B virus (HBV) is a significant contributor to the development of hepatocellular carcinoma (HCC). Chronic HBV infection (CHB) facilitates disease progression through various mechanisms. However, the specific factor responsible for the progression of HBV infection to HCC remains unresolved. This study aims to identify the hub gene linking CHB and HBV-related HCC through bioinformatic analysis and experimental verification.

**Methods:**

Differentially expressed genes (DEGs) were identified in datasets encompassing CHB and HBV-HCC patients from the GEO database. Enriched pathways were derived from GO and KEGG analysis. Hub genes were screened by protein–protein interaction (PPI) analysis and different modules in Cytoscape software. The significance of the selected hub gene in prognosis was further assessed in validated datasets. The effects of hub genes on cell growth and apoptosis were further determined in functional experiments.

**Results:**

The study revealed upregulation of NUSAP1 in CHBs and HBV-HCCs. High expression of NUSAP1 served as an independent predictor for poor prognosis of liver cancers. Functional experiments demonstrated that NUSAP1 promotes cell growth, influences cell cycle process, and protects cells from apoptosis in HepG2.2.15 cells.

**Conclusion:**

NUSAP1 serves as a poor prognostic indicator for liver cancers, and potentially plays a crucial role in HBV-HCC progression by promoting proliferation and inhibiting apoptosis.

**Supplementary Information:**

The online version contains supplementary material available at 10.1007/s12672-024-00922-4.

## Introduction

Hepatocellular carcinoma (HCC) is a prevalent and malignant cancer that is widely observed, ranking as the third leading cause of cancer-related deaths globally [1]. The tumorigenicity of HCC is multifaceted, with potential risk factors including viral infections, alcohol consumption, metabolic syndrome, and genetic mutations [2]. Notably, hepatitis B virus (HBV) infection is a major etiological factor closely associated with chronic hepatitis, cirrhosis, and HCC. Chronic HBV infection (CHB) is responsible for over 50% of HCC cases worldwide and 70–80% of HCC cases in regions with a high prevalence of HBV [3]. Advances in the study of the molecular processes behind HBV-induced HCC have uncovered a complicated landscape, which can be used to inform strategies for prevention, early detection, and post-operative care of this cancer. The primary pathways by which HBV contributes to the development of HCC are integration of its DNA into the host liver cell genome, which alters gene function, forms truncated proteins with carcinogenic potential and disrupts the host genome. Mutations in the S, C, and X regions of the HBV genome, activation of oncogenes by the HBx protein encoded by the HBV X gene, activation of tumor promoter genes and inhibition of tumor suppressor genes are also the reasons [4, 5].

Nucleolar spindle-associated protein 1 (NUSAP1) is a vital regulator of mitosis, weighing approximately 55 kDa, located on chromosome 15q15.1 [6]. It works to regulate spindle assembly and maintain normal mitosis by binding to microtubule and DNA domains, which is essential for chromatin-induced spindle formation, especially during early embryogenesis [7]. In recent years, researchers have discovered a link between NUSAP1 and cancer, high levels of NUSAP1 have been linked to the development, advancement, and unfavorable prognosis of various tumors, including esophageal squamous cell carcinoma [8], prostate cancer [9, 10], renal cell carcinoma [11] and glioma [12]. Previous research has demonstrated upregulation of NUSAP1 in HCC compared to non-tumor liver tissues [13], and that this upregulation promotes the proliferation and in vitro tumorigenicity of HCC cells. However, limited information is available regarding the expression and mechanism of NUSAP1 in HBV infection and liver cancer.

In this study, we conducted a more comprehensive bioinformatics analysis comparing normal liver tissues with chronic HBV-infected liver tissues, as well as HBV-HCC tissues with adjacent normal tissues. Our findings indicated a significant elevation in NUSAP1 expression in chronic HBV carriers and HBV-HCC tissues. Furthermore, knockdown of NUSAP1 inhibited the malignant biological behavior of liver cancer cells. Detecting these key targets early in both chronic hepatitis B infection and HBV-related HCC may be critical in halting the progression of HBV-related HCC during its initial stages.

## Materials and methods

### Dataset download and process

Six datasets were downloaded from the Gene Expression Omnibus (GEO) database (https://www.ncbi.nlm.nih.gov/geo/), including the gene expression profile data and related annotation files. The obtained datasets were screened by the following criteria: (1) Profile information encompassed both experiment and control groups. (2) All samples were derived from liver tissues and had not undergone chemoradiotherapy prior to surgical resection. (3) All datasets provided raw data suitable for further analysis. From these datasets, GSE121248, GSE83148 and GSE55092 were chosen to screen differentially expressed genes (DEGs). Additionally, GSE84044, GSE136247 and GSE65359 were chosen for subsequent validation.

### Identification of DEGs from HBV Infection to HBV-HCC

We used GEO2R (https://www.ncbi.nlm.nih.gov/geo/geo2r/) for preliminary comparison and downloaded the series matrix files of all the datasets from GEO datasets. The DEGs between experimental and control samples were identified using the “limma” package. Fold changes (FCs) were calculated for individual gene expression. DEGs were determined based on specific cutoff criteria of padj < 0.05 and |logFC|> 1.0. The DEGs was presented in volcano plot using “ggplot2” package, and in a heatmap using “pheatmap” package (Supplementary Fig. 1) respectively. The online Venn diagram tool jvenn (http://jvenn.toulouse.inra.fr/) was used to identify the common DEGs [14].

### Functional enrichment analyses of common DEGs

The “ClusterProfiler” package was utilized for conducting Gene Ontology (GO) and Kyoto Encyclopedia of Genes and Genomes (KEGG) pathway analyses. The GO analysis was divided into three categories: cellular component (CC), molecular function (MF), and biological process (BP), allowing examination of physiological functions.

The common significant DEGs were mapped to the STRING database https://cn.string-db.org/to construct a protein–protein interaction (PPI) network. For the PPI network analysis, a confidence level greater than 0.4 was set, and the network was visualized using Cytoscape software. Significant modules and hub genes were identified using the MCODE and CytoHubba. To further elucidate the biological features and mechanism of interacting proteins of the DEGs, functional annotation analysis was performed using another online software Metascape (http://metascape.org/).

### Validation of the chosen gene

The expression levels of the identified hub gene were validated in three GEO validation datasets. A comparison between the samples and controls was performed using the Wilcoxon test and a p value < 0.05 was considered significant.

Receiver operating characteristic (ROC) curves were generated to assess the predictive accuracy of the hub genes using the “pROC” package in the R language. To further validate the chosen gene, three online databases were utilized. The HPA database (https://www.proteinatlas.org/) provided information on tissue and cellular distribution of various human proteins using immunoassay technology. The Gene Expression Profiling Interactive Analysis (GEPIA) database (http://gepia.cancer-pku.cn/) was employed for validating gene expression. The HCCDB database (http://lifeome.net/database/hccdb/) served as a specific resource for Hepatocellular Carcinoma Diagnostic and Survival Analysis [15].

### Gene set enrichment analysis (GSEA)

Based on the median expression of NUSAP1, HBV-infected patients were divided into high- and low-expression groups. The WEB-based Gene Set Analysis Toolkit (http://www.webgestalt.org/) and the “cluster Profiler” package [16] were applied for GSEA analysis. GSEA analysis allowed us to examine functional categories, calculate enrichment scores of gene sets, and discover different functional phenotypes by comparing biological pathways between the two groups. Only KEGG pathways with false discovery rate (FDR) ≤ 0.05 were considered significant and included in the analysis.

### Assessment of the immune landscape

The Tumor Immune Estimation Resource (TIMER 2.0) (http://timer.cistrome.org/) was used to explore the correlation between NUSAP1 expression and immune cell infiltration.

To evaluate the immune infiltration of NUSAP1 in CHB and HBV-HCC patients, the R packages of “GSVA” were utilized to quantify the relative abundance of 16 immune cells and 13 immune-related pathways. Subsequently, we compared the enrichment scores across the groups and visualized the results using the “ggpubr” package.

### Cell culture and transfection

The human liver cancer cell lines HepG2 and HepG2.2.15 were provided from Wuxi Red Cross Blood Center with the original source being Cell Resource Center (IBMS, China). HepG2 and HepG2.2.15 cells were cultured in DMEM (Hyclone, (Hyclone, Logan, UT, USA) supplemented with 10% fetal bovine serum (FBS; Biological Industries, Israel) and 1% penicillin–streptomycin (P/S; Hyclone) at 37 ℃ in 5% CO_2_ environment.

The pHBV1.3 plasmid containing 1.3 copies of the HBV genome was kindly provided from Wuxi Red Cross Blood Center. The plasmid was originally purchased from Fenghui Biological Co., Ltd (Changsha, China). HepG2 cells were transfected with plasmids containing the HBV whole genome, while HepG2.2.15 cells were transfected with NUSAP1 siRNA using Lipofectamine3000 reagent (Thermo-Fischer Scientific, MA, USA) according to the manufacturer’s instructions. To authenticate the siRNA, we carried out three transfections and validated the results with three repeating trials.

si-NUSAP1_1: CCUUGAAAGGCUACAUUAATT;

si-NUSAP1_2: GGAAGACUCUCUGUGGCUUTT;

si-NUSAP1_3: CCAAGACUCCAGCCAGAAATT.

### Western blot and qRT-PCR

Liver cancer cells were lysed using RIPA buffer (Cell Signal Technology, MA), centrifuged for the supernatant. The protein concentration was measured using bicinchoninic acid (BCA) assay (Cwbio, Beijing, China). The lysates were then diluted in loading buffer and denatured by heating at 100 ℃. Standard Western blot assay were performed using NUSAP1 antibody (Proteintech, 12024-1-AP) and GAPDH antibody (Abcam, ab77109) as the loading control.

Total RNA was extracted from the liver cancer cells using Trizol reagent (Invitrogen, USA) and cDNA was synthesized using the M-MLV Reverse Transcriptase Kit (Cwbio) following the manufacturer’s instructions. RT-PCR was performed using Real SYBR Mixture (Cwbio) on a Lightcycler 480 II instrument (Roche Applied Science, USA). GAPDH severed as the internal control.

NUSAP1 forward primer: 5ʹ-AGCCCATCAATAAGGGAGGG-3ʹ;

NUSAP1 reverse primer: 5ʹ-ACCTGACACCCGTTTTAGCTG-3ʹ.

GAPDH forward primer: 5ʹ-TGTTGCCATCAATGACCCCTT-3ʹ;

GAPDH reverse primer: 5ʹ-CTCCACGACGTACTCAGCG-3ʹ.

### Cell counting kit-8 (CCK-8) analysis

HepG2.2.15 cells transfected with NUSAP1 siRNA were seeded in 96-well plates at a density of 1 × 10^3^/well for the specified duration. In total, 10 μL of CCK-8 reagent (CCK8; Vazyme, Nanjing, China) was added to each well and incubated for 2 h. The optical density (OD) at 450 nm was measured using a microplate reader (Thermo Fisher). The experiment was performed three times with six replicates.

### Cell cycle assay and apoptosis analysis

HepG2.2.15 cells were cultured for 24 h and then subjected to serum deprivation for 24 h to synchronize the cell cycle. Afterward, the cells were cultured in normal medium for an additional 24 h. Subsequently, the cells were harvested and fixed with 70% ethanol at − 20 ℃ for 24 h. For propidium iodide (PI) staining, the cells were incubated in the presence of 50 μg/mL propidium iodide (PI; Sigma-Aldrich, St Louis, MO) and 100 ng/mL RNase A (Boehringer Mannheim, Indianapolis, IN) at room temperature in the dark for 1 h. The cell cycle progression in each sample were measured via FACS Canto II (BD, Mountain View, CA). The percentages of cells in the G1, S, and G2 phases were calculated using ModFit LT software (RRID:SCR_016106, BD). Each experiment was repeated three times.

Cell apoptosis was detected by flow cytometry with Annexin V-FITC Apoptosis Detection Kit (KeyGen Biotech, China) according to the manufacturer’s instructions. Cells were incubated with 2 μM Doxorubicin (Dox, Slleck) for 48 h. The cells were then harvested, rinsed with ice cold PBS, and then resuspended in 200 μL of binding buffer. Next, 10 mL of AV-FITC stock solution was added to cell suspensions and incubated for 15 min at room temperature. Subsequently, the cells were rinsed with 200 μL of ice-cold PBS and immediately analyzed using a FACScan flow cytometry.

### Statistical analysis

Bioinformatics analysis was carried out using R software (version.4.2.1). Statistical significance was defined as p < 0.05 and FC > 1.5. In vitro experiments were repeated at least 3 times. Data were analyzed using SPSS 20.0 software (SPSS Inc., Chicago, IL, USA). p < 0.05 indicated that differences were statistically significant.

## Results

### Data information

A total of six GEO datasets including GSE83148, GSE55092, GSE121248, GSE84044, GSE136247 and GSE65359 were chosen for this study. Details regarding each dataset are summarized in Table 1. The discovery cohorts, consisting of GSE83148, GSE55092, and GSE121248, were selected for identifying common DEGs, while the remaining datasets were categorized as validation cohorts.

**Table 1:** Information of GEO datasets containing the chronic HBV infected patients and HBV-related HCC patients

Dataset	Platform	Samples	Controls	Disease	Group
GSE83148	GPL570	HBV-infected liver tissues (n = 122)	Healthy liver tissues (n = 6)	Chronic HBV infection	Discovery
GSE55092	GPL570	HBV-related HCC tissues (n = 49)	Adjacent normal tissues (n = 91)	HBV-induced HCC	Discovery
GSE121248	GPL570	HBV-related HCC tissues (n = 70)	Adjacent normal tissues (n = 37)	HBV-induced HCC	Discovery
GSE84044	GPL570	HBV-related CHB with fibrosis and inflammation (n = 43)	HBV-related CHB without fibrosis and inflammation (n = 26)	Chronic HBV infection	Validation
GSE136247	GPL17586	HBV-related HCC tissues (n = 26)	Adjacent normal tissues (n = 19)	HBV-induced HCC	Validation
GSE65359	GPL570	Immune clearance liver tissues (n = 50); Immune tolerant liver tissues (n = 22)	Inactive carriers' liver tissues (n = 11)	Chronic HBV infection	Validation

### Identification of DEGs throughout HBV-related liver diseases

**Fig. 1:**
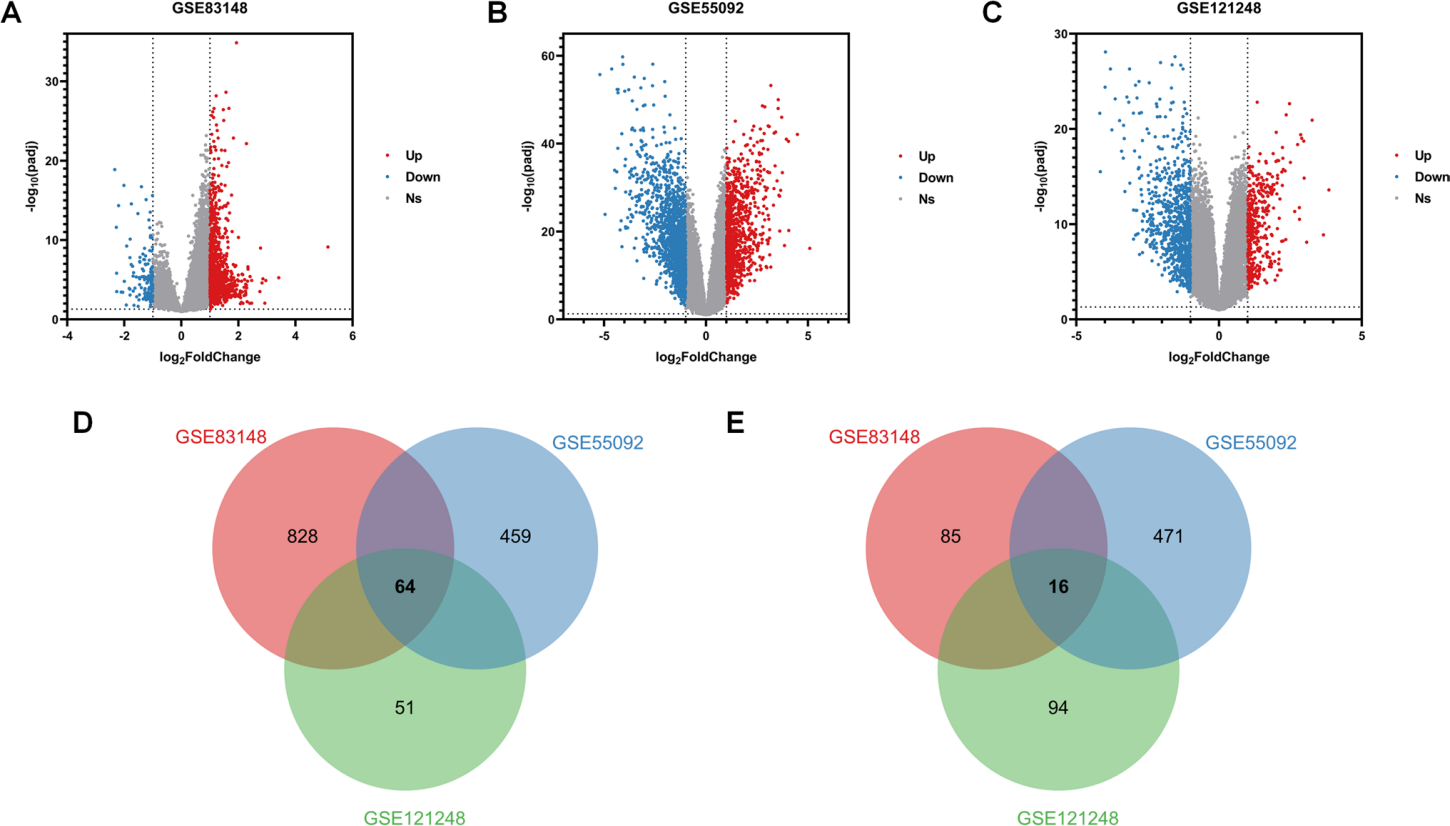
Identification of common DEGs from three GEO datasets. **A** Volcano plot displaying DEGs in GSE83148. **B** Volcano plot displaying DEGs in GSE55092. **C** Volcano plot displaying DEGs in GSE121248. In the volcano plots, red dots represent upregulated genes and blue dots represent downregulated genes. Venn diagram illustrates the overlap of upregulated **D** and downregulated genes **E** across the three datasets. *DEGs* Differentially Expressed Genes, *GEO* Gene Expression Omnibus

Using the “limma” package, we screened for DEGs between CHB patients and healthy controls in GSE83148. We found that 927 genes were upregulated and 113 genes were downregulated in this dataset (Fig. 1A). We further identified DEGs between HBV-related HCC tissues and adjacent normal tissues: 747 genes were upregulated and 1000 genes were downregulated in GSE55092; while 328 genes were upregulated and 625 genes were downregulated in GSE121248 (Fig. 1B, C). As illustrated in Fig. 1D, E, 80 common DEGs were identified across these three datasets, with 64 being upregulated and 16 being downregulated.

**Fig. 2:**
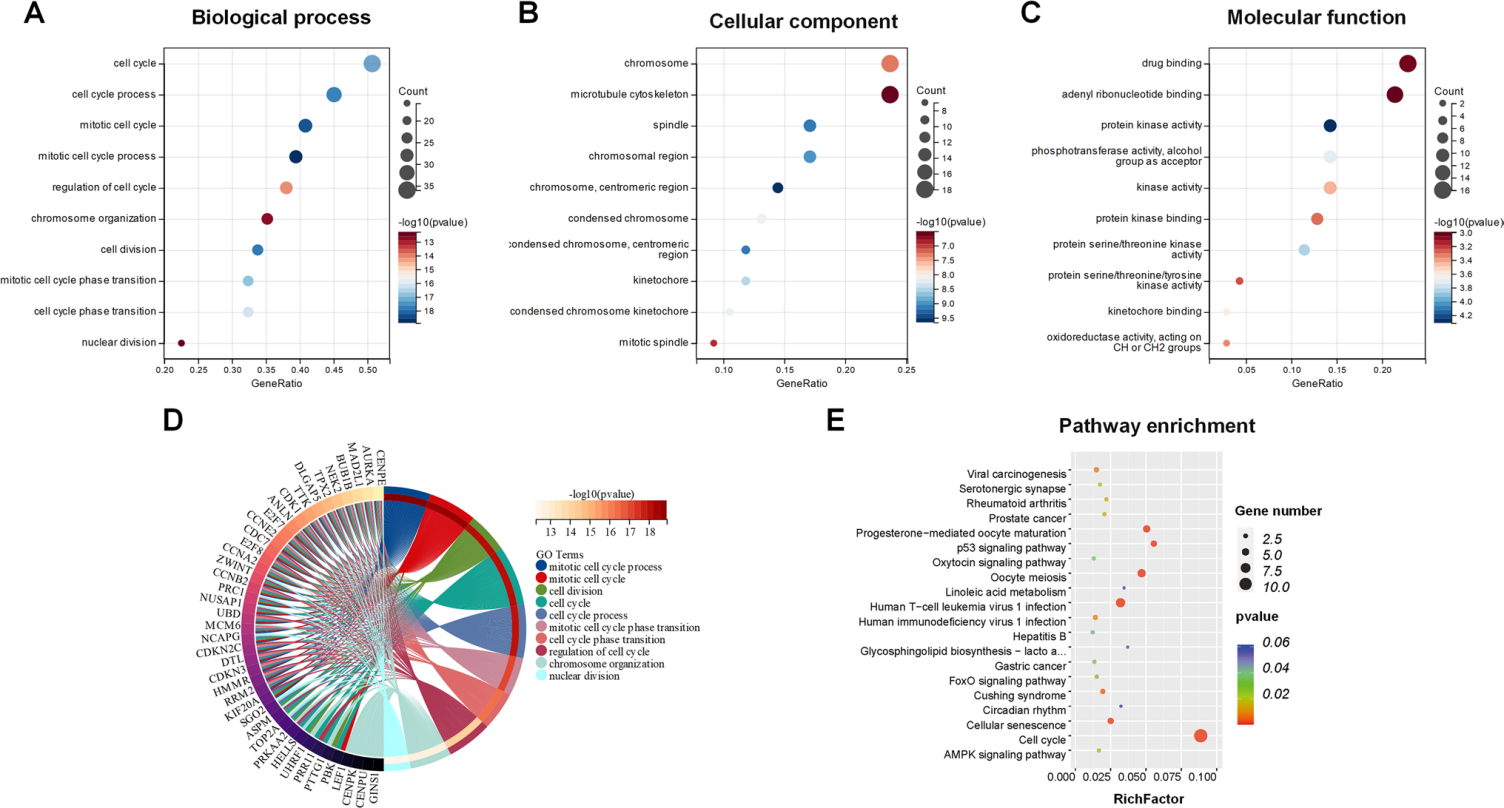
Functional enrichment analysis of 80 common DEGs from three datasets. **A** Bubble diagram illustrating the enriched biological processes in Gene Ontology (GO) analysis. **B** Bubble diagram depicting the enriched cellular component in GO enrichment analysis. **C** Bubble diagram representing the molecular function in GO enrichment analysis. **D** Chord plot visualizing the specific DEGs in GO analysis. **E** Bubble diagram displaying the enriched KEGG pathways for the 80 common DEGs. *GO* Gene Ontology, *KEGG* Kyoto Encyclopedia of Genes and Genomes

In order to understand the biological importance of 80 commonly observed DEGs, we conducted GO analysis and KEGG pathway enrichment. Our findings revealed that these genes were primarily associated with cell cycle processes in terms of biological functions. Additionally, they were found to be related to chromosome, microtubule cytoskeleton, and spindle in terms of cellular components. Furthermore, as for molecular function, the genes were mainly enriched in drug binding and protein kinase activity **(**Fig. 2A–C**)**. The GO analysis of specific DEGs revealed that they were closely associated with cell cycle activities, including nuclear division, chromosome organization, and cell cycle phase transition **(**Fig. 2D**)**. Additionally, the KEGG analysis indicated that the DEGs were mainly enriched in the cell cycle, virus infection, oocyte meiosis, and the p53 signaling pathway **(**Fig. 2E**)**.

**Fig. 3:**
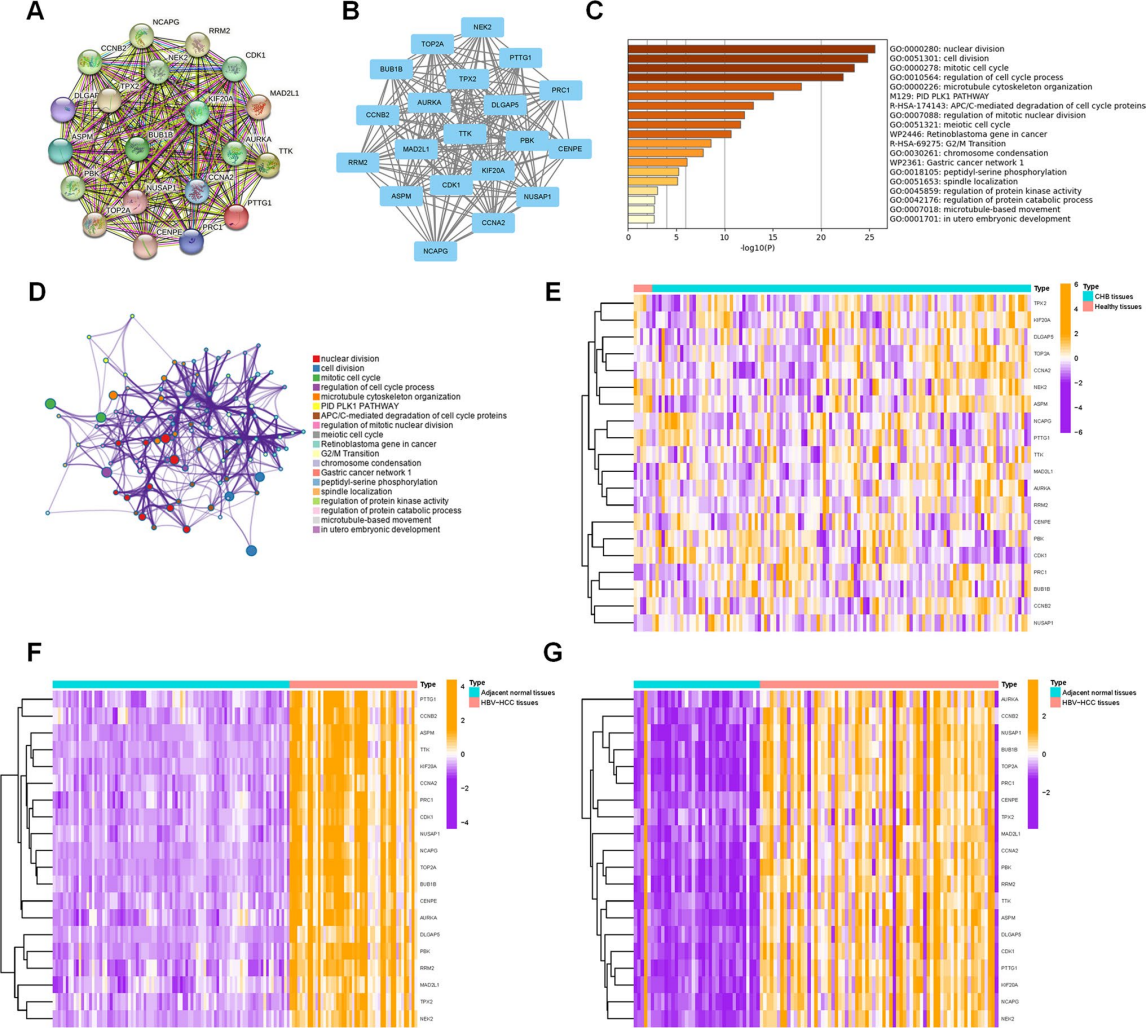
Identification of 20 hub genes using STRING and Metascape. **A** PPI network of the common DEGs constructed by STRING. **B** Significant gene module comprising 20 nodes and 183 edges, with a cluster score (density times the number of members) of 19.263. **C** GO and KEGG enriched terms colored according to p-values, as constructed by Metascape. **D** Network of GO and KEGG enriched terms colored according to clusters, as determined by Metascape. Heatmap displaying the expression of 20 hub genes in GSE83148 **E**, GSE55092 **F**, GSE121248 **G**. *PPI* Protein–protein interaction, *DEGs* Differentially Expressed Genes, *GO* Gene Ontology, *KEGG* Kyoto Encyclopedia of Genes and Genomes

To explore the potential connections among the proteins encoded by the commonly observed DEGs and identify key hub genes, we employed STRING to construct a protein–protein interaction (PPI) network for the 80 DEGs. Subsequently, module analysis was conducted using the MCODE plug-in in Cytoscape to identify significant clustering modules. As depicted in Fig. 3A, B, the most notable module consisted of 20 nodes and 183 edges. 20 candidate hub genes were selected from this module, including CENPE, TOP2A, NCAPG, PRC1, DLGAP5, PTTG1, TPX2, MAD2L1, TTK, AURKA, NEK2, PBK, CCNB2, NUSAP1, KIF20A, BUB1B, ASPM, RRM2, CCNA2 and CDK1.

Afterwards, the most significant module underwent GO analysis and KEGG pathway enrichment to investigate the underlying biological processes (Fig. 3C, D). The pathway enrichment results revealed that the gene function within this module was linked to protein binding, kinase activity, and cell cycle regulation, aligning with the biological function of common DEGs. Heatmaps of 20 candidate hub genes between CHB liver tissues and healthy tissues (Fig. 3E), between HBV-HCC tissues and adjacent normal tissues (Fig. 3F, G) were also illustrated to display their expression levels.

### Hub gene NUSAP1 and its prognostic value in HBV-related liver diseases

**Fig. 4:**
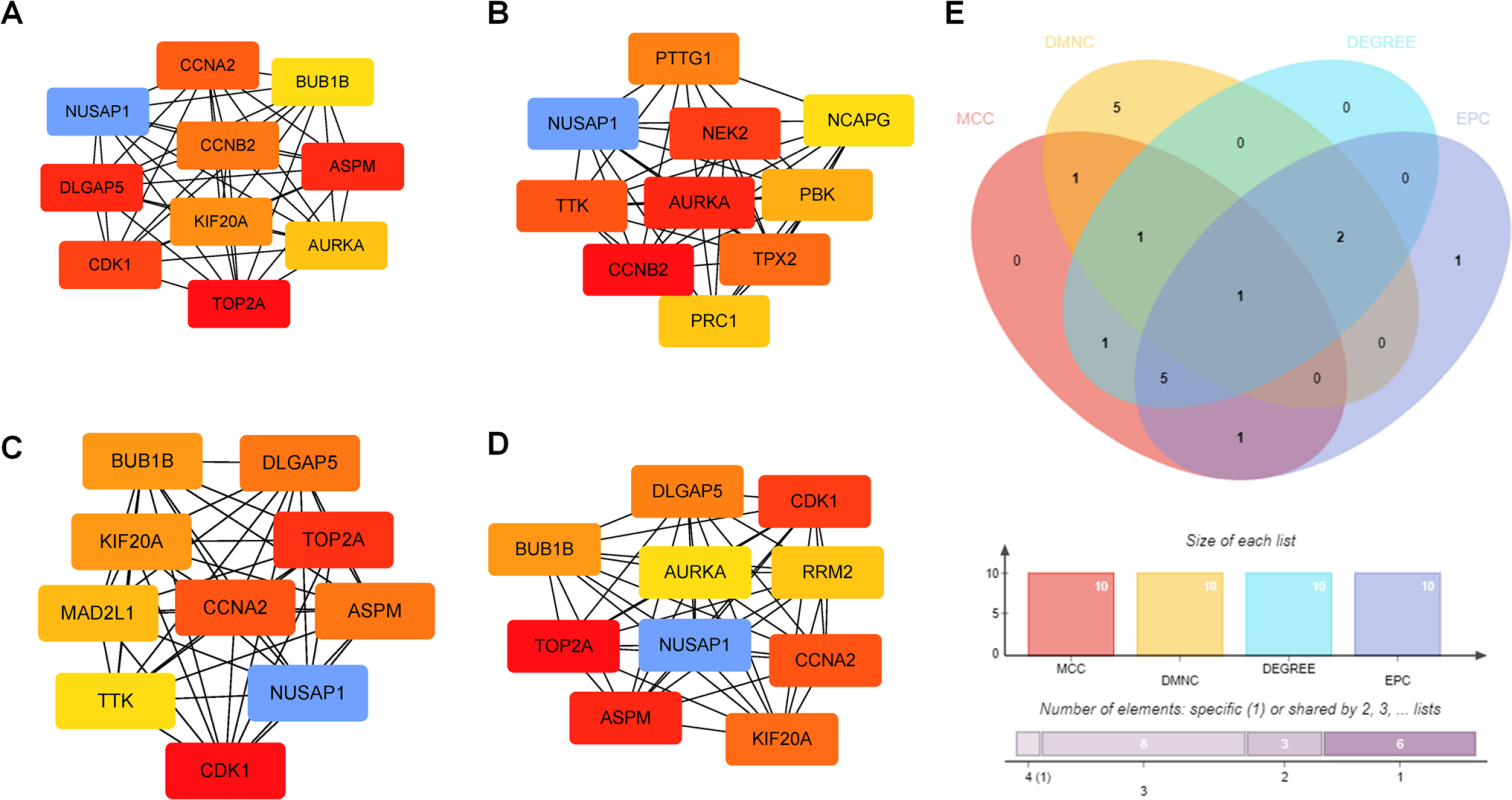
Selection of the most important target using four algorithms in Cytoscape. Four algorithms, namely **A** MCC (Maximal Clique Centrality), **B** DMNC (Degree-Maximum Neighbor Clique), **C** DEGREE, and **D** EPC (Edge Percolated Component), were applied to identify the top 10 important genes among the pool of 20 hub genes. **E** Venn diagram illustrating the identification of a candidate gene from the 20 hub genes using the four algorithms

**Fig. 5:**
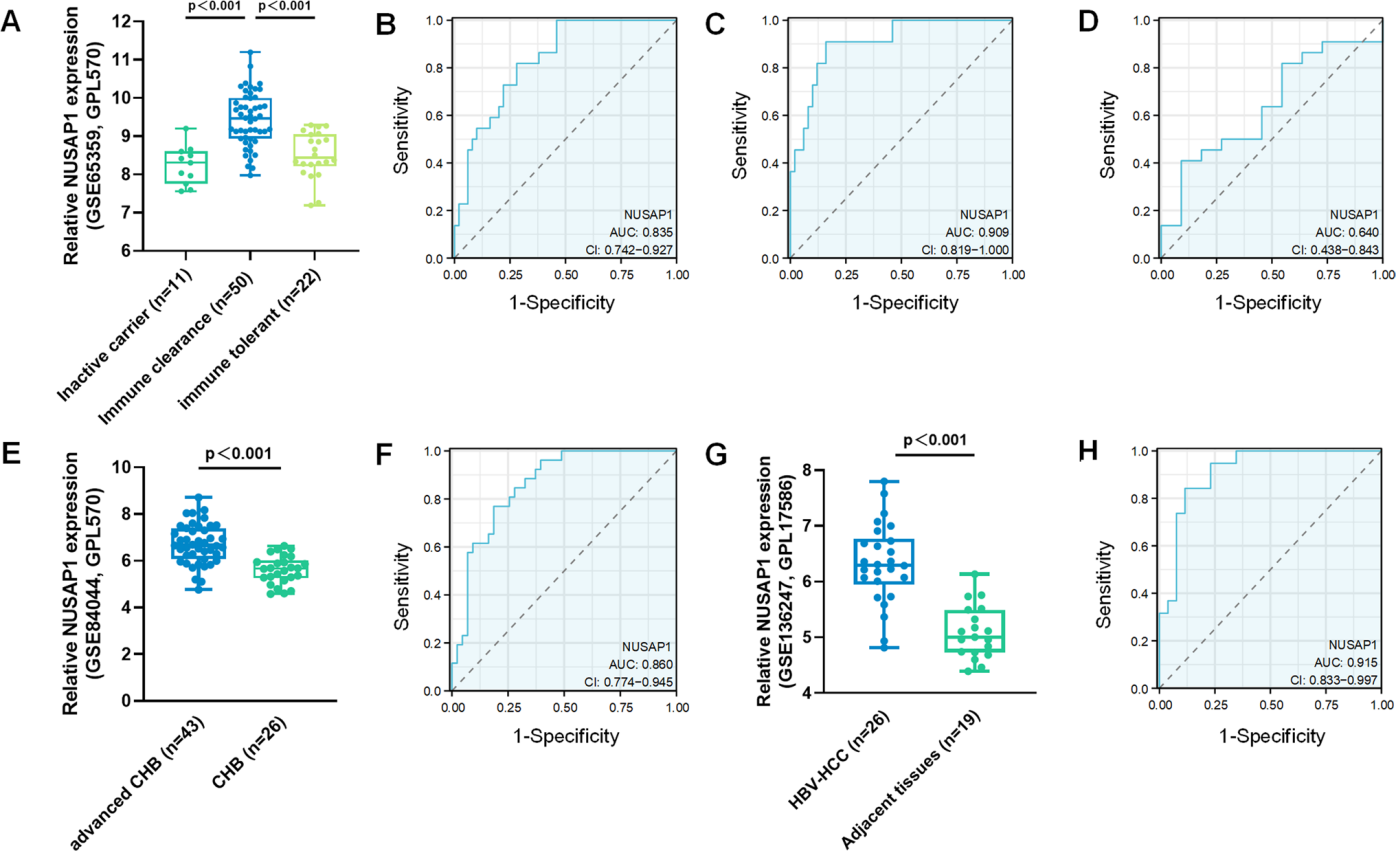
Expression and prognostic value (ROC curve) of NUSAP1 in validated datasets. **A** Expression of NUSAP1 across different HBV infection statuses in the GSE65359 dataset. **B** Potential diagnostic values of NUSAP1 between immune clearance and immune tolerant in the GSE65359 dataset. **C** Potential diagnostic values of NUSAP1 between immune clearance and inactive carriers in GSE65359. **D** Potential diagnostic values of NUSAP1 between immune tolerance and inactive carriers in GSE65359. **E**, **F** Expression and potential diagnostic values of NUSAP1 for advanced CHB with fibrosis and inflammation in the GSE84044 dataset; **G**, **H** Expression and potential diagnostic value of NUSAP1 for HBV-HCC and adjacent normal tissues in the GSE136247 dataset

In order to investigate the genes that may have a significant impact on the progression from CHB infection to HBV-HCC, we utilized CytoHubba to identify hub genes among the common DEGs. Given the heterogeneous nature of biological networks, we employed multiple topological analysis algorithms, namely MCC, MNC, Degree, and EPC, simultaneously to predict and explore the top ten crucial hub genes in the PPI networks. The convergence of these 10 genes from the four algorithms highlighted NUSAP1 as the most pivotal hub gene (Fig. 4). We downloaded GSE65359, GSE84044 and GSE136247 from the GEO database and analyzed NUSAP1 expression in different status of HBV-related liver diseases. The expression of NUSAP1 were elevated in immune clearance status, compared to inactive carriers and immune tolerant status. Moreover, NUSAP1 showed great accuracy for the diagnosis of HBV-related immune clearance status (Fig. 5A–D). In advanced CHB status with fibrosis and inflammation, NUSAP1 exhibited higher expression levels compared to CHB status without advanced symptoms and could differentiate between CHB with and without fibrosis and inflammation with AUC_NUSAP1_ = 0.860 (Fig. 5E, F). The expression level of NUSAP1 was also higher in HBV-HCC tissues and effective in distinguishing HBV-HCC tissues from adjacent normal tissues with AUC_NUSAP1_ = 0.915 (Fig. 5G, H).

**Fig. 6:**
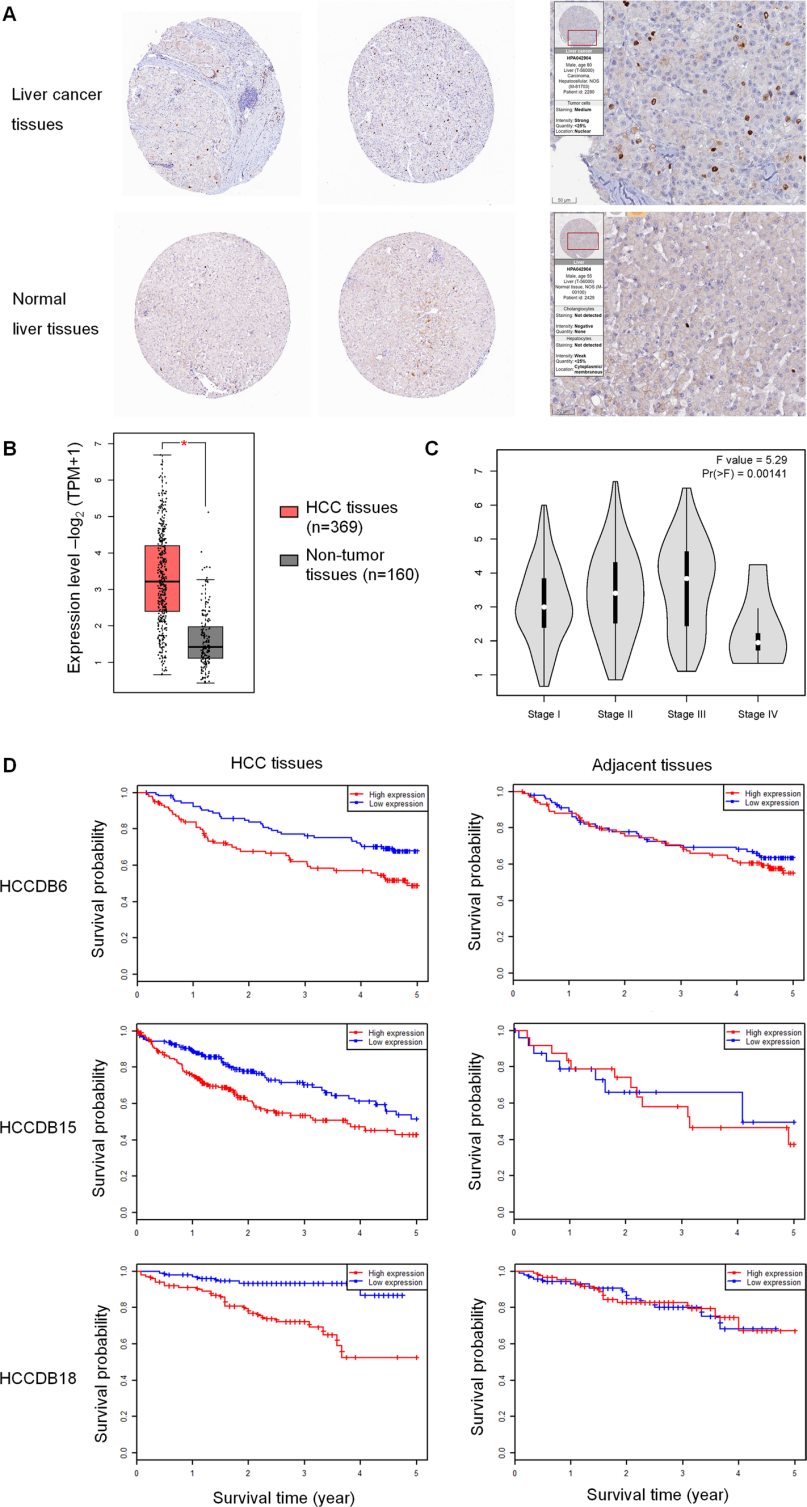
Expression, location and prognosis value of NUSAP1 in different databases. **A** Expression and location of NUSAP1 in liver cancer tissues and normal liver tissues in the HPA database. **B**, **C** NUSAP1 expression data from the GEPIA database. **D** Survival probability in NUSAP1-high and NUSAP1-low expression group in the HCCDB database, including three datasets: HCCDB6, HCCDB15 and HCCD818. *p < 0.05

Using the HPA websites, we predicted the intracellular localization of NUSAP1 proteins. Compared to normal liver tissues, the NUSAP1 proteins were found to be located in the cell nucleus with strong intensity in HCC tissues (Fig. 6A). In GEPIA, the expression level and prognosis value of NUSAP1 in HCC patients based on LIHC data from TCGA were analyzed. More NUSAP1 were expressed in LIHC tumor samples. From stage I to stage III, the expression of NUSAP1 was gradually increased (Fig. 6B, C). In Fig. 6D, the Kaplan–Meier survival analysis of three HCCDB databases displayed higher levels of NUSAP1 in HCC tissues associated with poor overall survival among HCC patients, while NUSAP1’s expression in adjacent normal tissues didn’t correlate with survival probability.

### Gene set enrichment analysis identified NUSAP1-associated signaling pathways

**Fig. 7:**
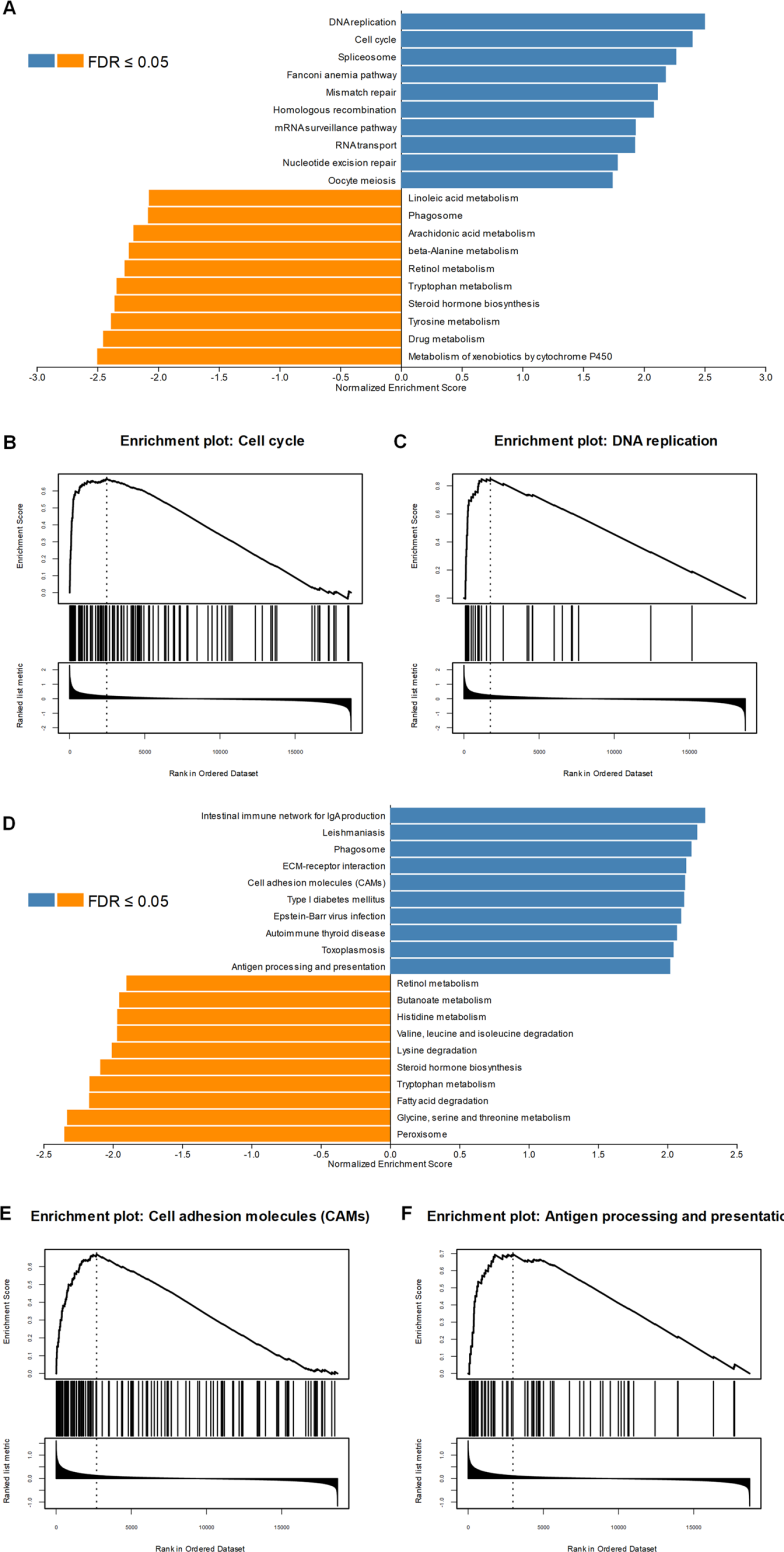
Enriched pathways in HBV-HCC and CHB patients with NUSAP1-high expression. **A** Top 10 enriched pathways in HBV-HCC patients with NUSAP1-high expression; GSEA analysis illustrated that NUSAP1 overexpression might be involved in cell cycle progression (**B**) and DNA replication process (**C**). **D** Top 10 enriched pathways in CHB patients with NUSAP1-high expression; GSEA analysis illustrated that NUSAP1 overexpression might be involved in cell adhesion molecules (**E**) and antigen processing and presentation (**F**). *GSEA* Gene Set Enrichment Analysis

GSEA analysis was conducted to identify distinctively regulated pathways between the high and low expression groups of NUSAP1, as well as to determine the activated signaling pathways in liver diseases associated with HBV. Activated pathways including DNA replication and cell cycle were found in HBV-HCC patients with overexpressed NUSAP1 (Fig. 7A, C). In CHB patients with high NUSAP expression, the enriched pathways were different from HBV-HCC patients and were associated with cell adhesion molecules, antibody production and antigen presentation (Fig. 7D, F). Our findings suggested that inhibiting the activation of pathways that are distinct from classic tumor-associated signaling pathways may hold potential for reversing the progression of chronic hepatitis.

### Association between NUSAP1 and the tumor microenvironment

**Fig. 8:**
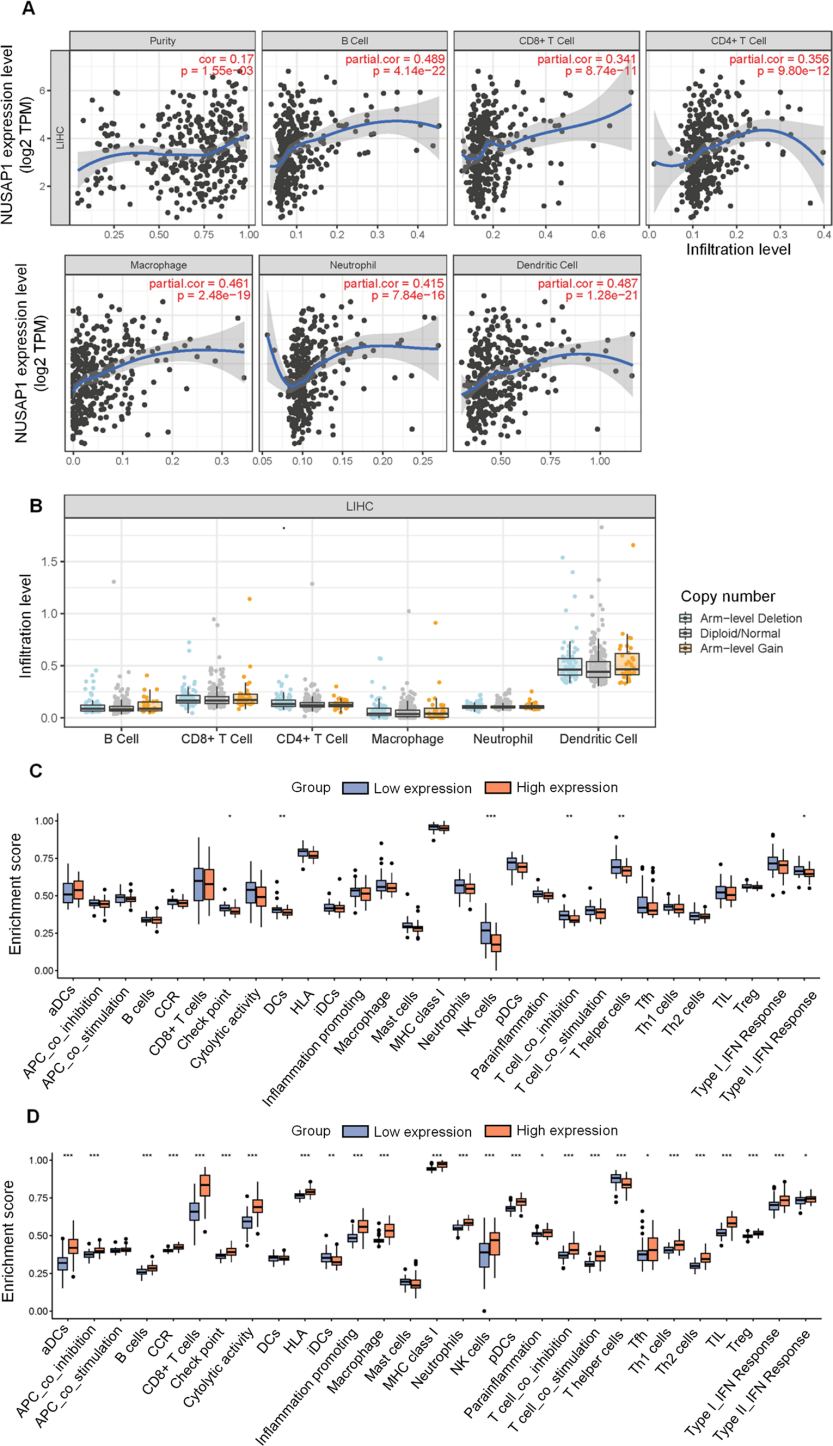
Association Between NUSAP1 and the Tumor Microenvironment. **A** Correlation between NUSAP1 expression and immune cells infiltration from TIMER. **B** Relationship between somatic copy number variation of NUSAP1 and immune infiltration as determined by TIMER. **C** Differences in ssGSEA score of 29 immune pathways between the high and low NUSAP1 expression groups in GSE121248. **D** Differences in ssGSEA score of 29 immune pathways between the high and low NUSAP1 expression group in GSE83148. Statistical significance: *p < 0.05, **p < 0.01, and ***p < 0.001. ssGSEA, single sample Gene Set Enrichment Analysis

According to TIMER database, NUSAP1 expression was positively correlated with B cells, CD8^+^T cells, CD4^+^T cells, macrophages, neutrophils, and dendritic cells (Fig. 8A). Specifically, a higher number of somatic copy number variations of NUSAP1 was associated with increased dendritic cell infiltration (Fig. 8B). Furthermore, when HBV-HCC patients were categorized based on NUSAP1 expression, the high and low NUSAP1 expression groups showed significant differences in the ssGSEA (single sample Gene Set Enrichment Analysis, ssGSEA) scores of 29 immune pathways (Fig. 8C). Similarly, in CHB patients, dividing them based on NUSAP1 expression revealed significant differences in the activation of immune pathways, indicating heightened immune signaling during inflammatory infections (Fig. 8D). These findings suggest that more immune pathways are activated in the inflammatory period, while some pathways may be inhibited during the tumor progression.

### Inhibition of NUSAP1 suppressed HepG2.2.15 proliferation and increased apoptosis

**Fig. 9:**
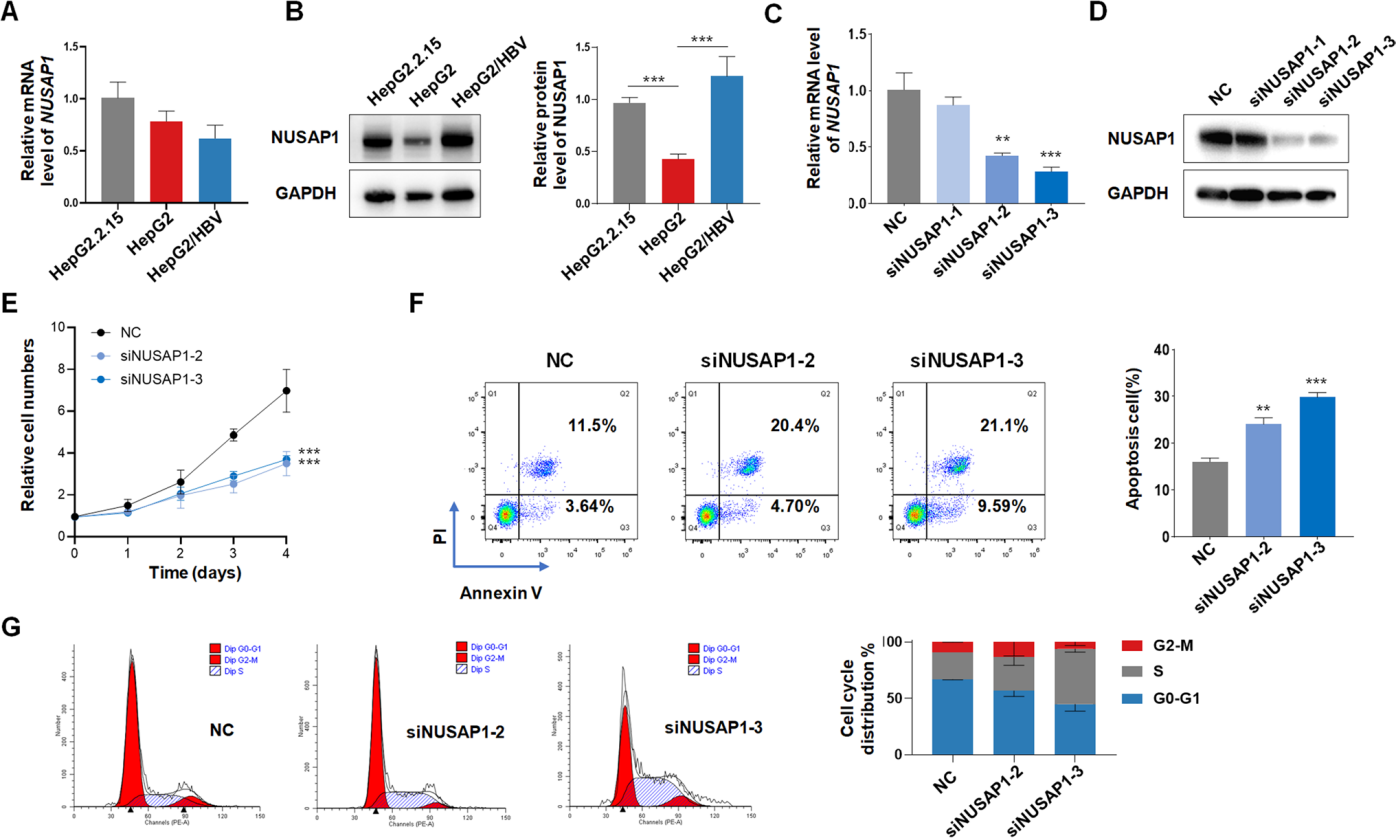
Effects of NUSAP1 knockdown on HBV-HCC cell proliferation, apoptosis, and cell cycle. **A** qRT-PCR analysis of NUSAP1 mRNA expression in HepG2-derived/related HCC cells. **B** Western blot analysis of NUSAP1 expression in HepG2-derived/related HCC cells. **C** qRT-PCR analysis and **D** Western blot analysis of NUSAP1 expression in HepG2.2.15 cell transduced with siRNA. **E** Cell proliferation assay (CCK-8) in transduced HepG2.2.15 cell. **F** Analysis of cell apoptosis in HepG2.2.15 cells with NUSAP1 knockdown. **G** Cell cycle analysis of HepG2.2.15 cells with NUSAP1 knockdown. Statistical significance: * p < 0.05 ∗ p < 0.01, and  ∗  ∗ p < 0.001

We compared the expression of NUSAP1 at both mRNA and protein levels in HepG2.2.15, HepG2 and HepG2 transfected with plasmid containing HBV whole genome (HepG2/HBV). The results indicated that after transfection with HBV plasmids, there was no variation in the RNA level of NUSAP1, but the protein level increased (Fig. 9A, B). Similarly, we transfected the HBV plasmid in Huh7 cells and noticed no dissimilarity in the RNA level of NUSAP1, yet the protein level decreased (Supplementary Fig. 2A, B). We selected HepG2.2.15 for functional validation experiments, which showed a relatively high expression of NUSAP1. We knockdown NUSAP1 using three siRNAs, siNUSAP1-1 didn’t show any effect (Fig. 9C, D), thus we continue the study using the siNUSAP1-2 and siNUSAP1-3 site, the proliferation ability of HepG2.2.15 was significantly reduced (Fig. 9E), as well as a decrease in colony number was observed in the NUSAP1 knockdown cells (Supplementary Fig. 3), suggesting that NUSAP1 may promote cancer cell proliferation. Annexin V/PI staining and flow cytometry were used to detect apoptosis in NUSAP1 knockdown HepG2.2.15 cells, revealing a significant increase in the proportion of cells undergoing apoptosis in the NUSAP1 knockdown group (Fig. 9F). Flow cytometry analysis after PI staining revealed that NUSAP1 knockdown HepG2.2.15 cells were arrested in the S phase of the cell cycle (Fig. 9G). Our findings suggested that NUSAP1 may promote cancer cell proliferation, influence cell cycle related pathways, and inhibit cell apoptosis, thereby contributing the survival of tumor cells.

## Discussion

CHB is a major cause of viral hepatitis, cirrhosis, and HCC. From HBV infection to the occurrence of HCC, HBV plays an important role either independently or synergistically with a variety of carcinogenic factors. Numerous studies have been conducted to uncover the potential mechanisms of HBV in the development and prognosis of liver cancer.

In this research, we employed diverse datasets from the GEO database to identify potential hub genes associated with HBV-HCC. We first identified shared DEGs in both CHB and HBV-HCC. Subsequently, we carried out protein–protein interaction and pathway enrichment analyses to determine the signal pathways enriched in HBV-related liver diseases. Finally, using different modules from STRING software, we identified a key gene, NUSAP1, which may have a significant impact on the transition from HBV infection to HCC progression.

Mitotic dysregulation has emerged as a potential indicator of malignancy. NUSAP1, an essential protein that binds to microtubules and chromatin, primarily localizes in the nucleolus, and participates in chromosome segregation and spindle assembly during mitosis. It stabilizes and cross-links microtubules, which leads to the formation of microtubule bundles and control of chromosome movement. Additionally, it regulates the dynamics of mitochondrial microtubules. Consequently, NUSAP1 has a pivotal role in mitosis and contributes to the development and prognosis of various cancers. Notably, down-regulation of NUSAP1 expression has been observed to inhibit migration, proliferation, and invasion of renal cancer cells [11], whereas high expression of NUSAP1 has been found to promote the growth and invasion of prostate cancer cells [9]. In breast cancer, NUSAP1 has been associated with poor overall survival and infiltration of various immune cells [17]. Moreover, in HCC, increased NUSAP1 expression has been linked to a poor prognosis, particularly early recurrence, and has been shown to facilitate early recurrence of HCC [18].

There is a lack of literature on the correlation between HCC and NUSAP1 expression. This study aims to investigate the relationship between HBV infection, NUSAP1 expression, and HCC. Our findings indicate that elevated NUSAP1 expression is linked to progressive liver diseases, immune cell infiltration and poor prognosis of HCC. Furthermore, inhibiting NUSAP1 expression impeded the growth of HBV-integrated HCC cells in vitro. A considerable number of HCC cells were arrested in the S phase of DNA replication and could not proceed to G2/M phase, which is the primary function of NUSAP1. NUSAP1 knockdown also induced cell apoptosis, highlighting the pivotal role of NUSAP1 in HBV-HCC development.

The development of HCC is a complex and multifaceted process. NUSAP1 may play a role in the progression from HBV infection to liver cirrhosis, ultimately leading to liver cancer. Our investigation into the localization of NUSAP1 revealed that in HCC tissues, NUSAP1 primarily resided in the nucleus, whereas in normal liver tissues, it was predominantly found in the cytoplasm (Fig. 6). Previous studies have shown that the dynamic localization of NUSAP1 is associated with the cell division cycle [6]: Specifically, during interphase and telophase of mitosis, NUSAP1 is located in the nucleolus in its dephosphorylated form, which represents its active state, indicating its role in the nucleus. Conversely, when NUSAP1 is present in the cytosol, it is phosphorylated and rendered inactive. Based on our functional studies, which showed that NUSAP1 knockdown inhibited cell growth, we hypothesize that NUSAP1 is constitutively expressed in the nucleus, promoting malignant biological behavior of HCC cells by accelerating the cell cycle and facilitating extensive cell proliferation.

In contrast to previous studies that focused on established tumors, our analysis incorporated the dataset of CHB patients and examined them. We observed a significant increase in NUSAP1 expression in liver tissues from patients with both CHB and HCC. Additionally, our validation datasets revealed that NUSAP1 expression was higher in CHB patients with inflammation and cirrhosis. Upon analyzing the immune infiltration in patients with CHB and HBV-HCC, we discovered that the high NUSAP1 expression group exhibited greater infiltration of immune cells. In CHB patients, a wider range of immune cells was observed to infiltrate. Our hypothesis is that this may be linked to the reported biological function of NUSAP1 in enhancing tumor stemness [18, 19]. NUSAP1 knockout decreased the expression of surface markers of cancer stem cells (CSCs), and NUSAP1 was highly expressed in self-renewing CSCs. CSCs can acquire the ability to evade the immune system and generate an immunosuppressive microenvironment, thereby promoting tumor recurrence and drug resistance [19]. In our study, we observed a greater infiltration of immune cells in CHB patients with high NUSAP1 expression, while the types of immune cells infiltrated in HBV-related HCC were reduced. This may be due to the role of NUSAP1 in promoting immune evasion of CSCs during the progression of liver disease.

To further investigate the cancer-promoting mechanism of NUSAP1, we focused on identifying additional therapeutic targets by examining both upstream and downstream regulators of NUSAP1. Previous studies have reported that NUSAP1 transcription can be positively regulated by transcription factors c-myc, LIN9, and NF-Y [20], as well as the E2F1/RB1 axis [9, 21]. Additionally, studies on non-coding RNA have revealed that miR-193a-5p targets NUSAP1 and its low expression in HCC can lead to increased NUSAP1 expression, which is associated with poor patient prognosis [22]. In glioma, the long non-coding RNA LINC01393 promotes the malignant biological behavior of tumors by regulating the miR-128-3p/NUSAP1 axis [23]. Furthermore, miR-18b mediates the regulation of NUSAP1 by HBV viral proteins. In vivo and in vitro experiments have demonstrated that miR-18b inhibits NUSAP1 and further suppresses the proliferation of HCC cells. The HBV x protein (HBx) can bind to NUSAP1 mRNA through miR-18b and inhibit miR-18b to maintain high NUSAP1 expression, thereby promoting HCC cell proliferation [24]. HBx inhibits miR-18b to maintain high expression of NUSAP1 and further promotes the proliferation of HCC cells. HBx is a crucial oncogenic protein in the process of HBV infection, and the specific role of other HBV viral proteins and NUSAP1 remains to be elucidated.

The downstream regulatory pathways of NUSAP1 have been extensively studied. Down-regulation of NUSAP1 can effectively inhibit the growth and metastasis of non-small cell lung cancer cells by regulating the BTG2/PI3K/Akt signaling pathway [25], while up-regulation of NUSAP1 can promote epithelial-mesenchymal transition through the Wnt/β-catenin signaling axis, thereby regulating the invasion and metastasis of triple-negative breast cancer cells [26]. NUSAP1 has also been shown to participate in epithelial-mesenchymal transition by regulating FAM101B, an effector molecule of the TGFβ1 signaling pathway, thus promoting the invasion, migration, and metastasis of prostate cancer [27]. Additionally, NUSAP1 can regulate DNA damage repair and a series of activities involving transcription, cellular stress, and DNA repair by binding to ILF2 [10]. NUSAP1 expression can also lead to increased invasion of astrocytoma cells by activating the Hedgehog signaling pathway [28]. In the recurrence of HCC, NUSAP1 can enhance tumor stemness by stimulating STAT3 nuclear translocation and RACK1 activation [18]. Further studies on the downstream signaling pathways of NUSAP1 will help to elucidate the specific mechanisms by which NUSAP1 promotes cancer and identify additional therapeutic targets for cancer treatment.

Our study has several limitations. Firstly, we acknowledge the importance of the prolonged stage of chronic hepatitis and cirrhosis in individuals infected with CHB before the development of HCC. However, we did not provide a clear explanation of the specific role that NUSAP1 plays in this prolonged process. Further investigation is needed to understand the involvement of NUSAP1 in the progression from chronic hepatitis to HCC. Secondly, we observed that NUSAP1 has a carcinogenic effect in HCC, and that HBV may accelerate this effect, the regulatory mechanisms by which HBV influences NUSAP1 expression are not fully understood. Finally, while the downstream regulatory pathways of NUSAP1 have been extensively studied in various types of tumors, the specific regulatory mechanisms of NUSAP1 in promoting the progression of HBV-HCC require further exploration and verification through additional clinical samples.

In conclusion, our findings suggest that NUSAP1 plays a crucial role in the progression of HBV infection, serving as a significant risk factor for HCC and a potential therapeutic target for HBV treatment. We believe that our findings will provide new insights and approaches to the accurate management of CHB and HBV-HCC, while also advancing our understanding of the carcinogenic mechanism involving NUSAP1.

### Supplementary Information

**Additional file1** Additional Figures, **Figures S1-S4**.

**Additional file2** Additional Table. Data from GSE3526 and GSE83148 indicate the differentially expressed genes (DEGs)in HBV-infected liver tissues compared with healthy controls.

**Additional file3** Additional Table. Data from GSE55092 and GSE121248 indicate the DEGs in HBV-HCC tissues comparedwith adjacent normal tissues.

**Additional file4** Additional Table. The common DEGs of both HBV-infected liver tissues and HBV-HCC tissues.

## Data Availability

All the datasets in this study can be downloaded from public databases: GEO (https://www.ncbi.nlm.nih.gov/geo/). The bioinformatics data are available from the corresponding author on reasonable request.
