# Supplementary material for: Unveiling NUSAP1 as a common gene signature linking chronic HBV infection and HBV-related HCC
Source: Discov Oncol. 2024 Mar 5;15:61. doi: 10.1007/s12672-024-00922-4 (PMC10914659; doi:10.1007/s12672-024-00922-4)

**Unveiling NUSAP1 as a Common Gene Signature Linking Chronic HBV Infection and HBV-Related HCC**

**Supplementary Figure 1. Heatmap of differentially expressed genes in three datasets GSE83148 (A), GSE55092 (B), GSE121248 (C).**


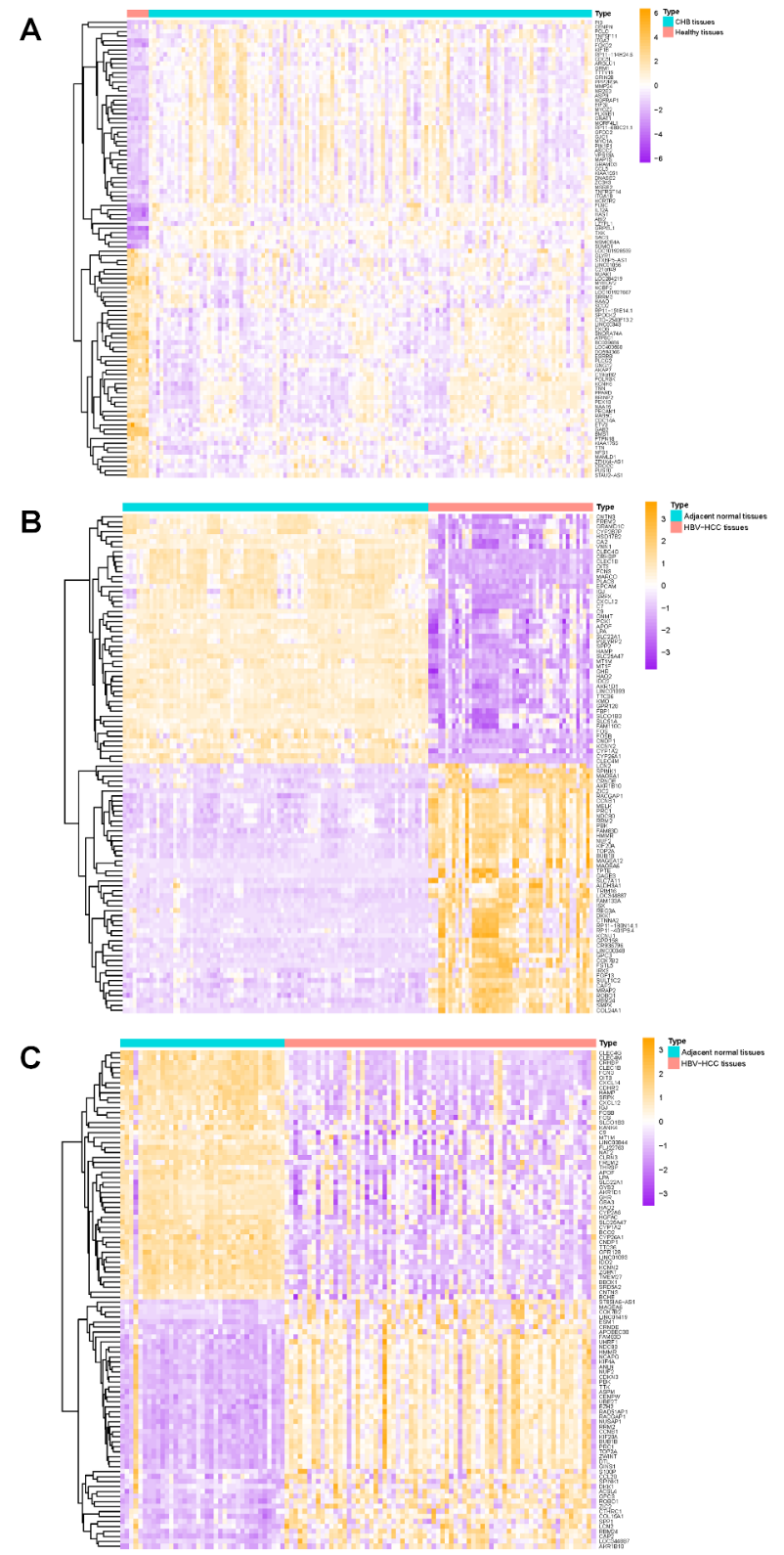


**Supplementary Figure 2. Effects of NUSAP1 on HBV-Huh7 cell. (A)** qRT-PCR analysis of *NUSAP1* mRNA expression in Huh7 and Huh7/HBV cells. **(B)** Western blot analysis of NUSAP1 expression in Huh7 and Huh7/HBV cells.


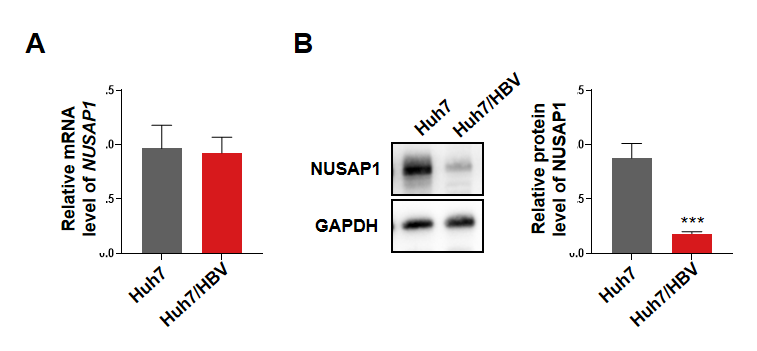


**Supplementary Figure 3. Colony formation in NUSAP1 knockdown HepG2.2.15 cells**

**
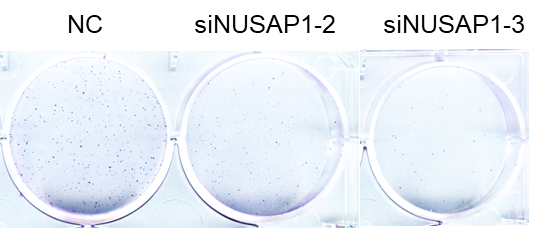
**

**Supplementary Figure 4. Original Western blot image of Figure 9B and 9D.**


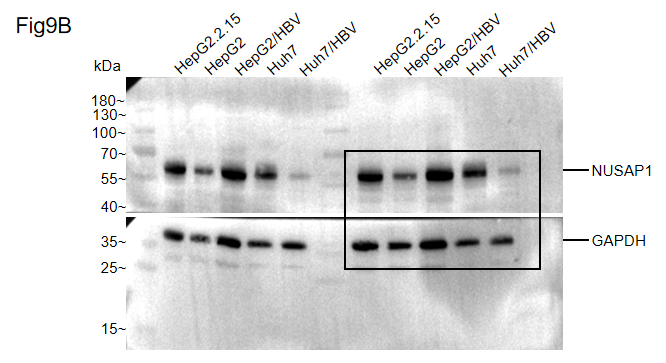


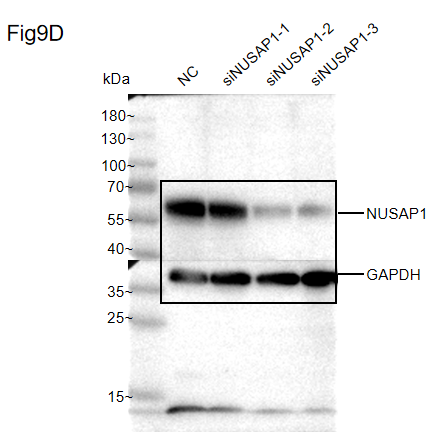

Supplement: Supplementary file 1 — Additional file1 Additional Figures, Figures S1-S4. [file 12672_2024_922_MOESM1_ESM.docx]
